# Trans-Carotid and Trans-Radial Access for Mechanical Thrombectomy for Acute Ischemic Stroke: A Systematic Review and Meta-Analysis

**DOI:** 10.7759/cureus.8875

**Published:** 2020-06-28

**Authors:** Aleka N Scoco, Aravind Addepalli, Shaoyu Zhu, Joshua Benton, Santiago R Unda, Neil Haranhalli, Richard Zampolin, David D Pasquale, Allan Brook, David Altschul

**Affiliations:** 1 Neurological Surgery, Montefiore Medical Center, New York, USA; 2 Neurological Surgery, Montefiore Medical Center, Bronx, USA; 3 Radiology, Montefiore Medical Center, New York, USA; 4 Neuroradiology/Neurological Surgery, Albert Einstein College of Medicine, Montefiore Medical Center, Bronx, USA

**Keywords:** transradial, transcarotid, acute ischemic stroke, thrombectomy

## Abstract

Objective

We aim to demonstrate the safety and effectiveness of extra-femoral endovascular access for mechanical thrombectomy for acute ischemic stroke patients whose vascular anatomy precludes safe or maneuverable trans-femoral access.

Methods

Preferred Reporting Items for Systematic Reviews and Meta-Analyses (PRISMA) guidelines were used to conduct a systematic review and meta-analysis with articles published until March 2018. The search protocol, including research questions and inclusion and exclusion criteria, were developed a priori. Our own institutional retrospective data were included in the cohort of case series.

Results

Eleven studies including 51 patients were included. Age ranged from 4th to 10th decade of life (average: 9.3rd decade) and 40.1% received IV tissue plasminogen activator. Initial National Institutes of Health Stroke Scale (NIHSS) score ranged from 1 to 36, (average: 17.6). Of the 51 patients, 39 (76%) patients suffered from anterior circulation large vessel occlusions versus 12 (24%) from posterior circulation occlusions. Site of access included 26 (51%) radial artery punctures, 23 (45%) direct percutaneous cervical carotid punctures, 1 brachial artery puncture, and 1 direct extradural vertebral artery puncture. Technical success was achieved in 43/51 (84%) of patients. The average modified Rankin Scale at discharge was 2.93 (n=26). There were no complications in 25 patients who underwent radial arterial access. Two (7.4%) of 27 cervical access patients developed hematoma.

Conclusions

Trans-carotid and trans-radial access for intervention in acute ischemic stroke is safe and effective. There may be instances in which these approaches should be considered first line before standard femoral approaches.

## Introduction

Mechanical thrombectomy has become the standard of care for acute stroke patients with large vessel occlusion (LVO). In all subset analyses of the randomized controlled trials (RCTs), time from presentation to vessel recanalization remains a critical element in improving functional outcomes [1]. As practice moves away from randomized trials into the real world of patient care, there are subsets of patients in whom standard trans-femoral access (TFA) is difficult or unachievable, or increases time to recanalization as compared with other potential routes such as trans-radial and trans-carotid. We explored the past literature and our own personal perspective.

In this case series and meta-analysis, we aim to demonstrate the safety and effectiveness of extra-femoral endovascular access for mechanical thrombectomy for acute ischemic stroke (AIS) patients whose vascular anatomy precludes safe or maneuverable TFA.

## Materials and methods

A systematic review and meta-analysis were performed in accordance with the Preferred Reporting Items for Systematic Reviews and Meta-Analyses (PRISMA) guidelines. The search protocol, including research questions and inclusion and exclusion criteria, were developed a priori. A literature search was performed using relevant key words to identify thrombectomy cases that used radial or cervical access. Articles were identified through the Ovid Medline and Web of Science databases from inception to March 2018, as well as our center’s unpublished data (named as Altschul's cases). There were no relevant Cochrane database studies.

Only studies dedicated to patients undergoing thrombectomy for ischemic stroke were included. The references of included publications were searched manually for other relevant papers. The following key words were used in combination: “stroke,” “endovascular,” “interventional,” “embolectomy,” “thrombectomy,” “cervical access” or “radial access”. We also reviewed references of key articles. Following informed consent exemption by the institutional review boards, retrospective data were collected of all patients who received endovascular mechanical thrombectomy for AIS patients through June 2018. Study authors were not contacted to obtain incomplete or unpublished data. Inclusion criterion was primarily focused on the relevant keywords as well as included data detailing the location of vessel occlusion, location of alternative access, and procedural outcome for the patient (thrombolysis in cerebral infarction [TICI] score). Other outcome measures, such as modified Rankin Scale (mRS), time from symptom onset to first pass and from access to recanalization, initial attempt to femoral access prior to alternative access, and other patient demographics, were included when available but did not comprise exclusion criteria for this study.

STATA 12 software (StataCorp., College Station, TX, USA) was used for statistical analysis, and results are shown as mean with 95% confidence interval calculated for all average data points.

Case descriptions

Two representative case descriptions from our unpublished data are included below

Case 1

**Figure 1:**
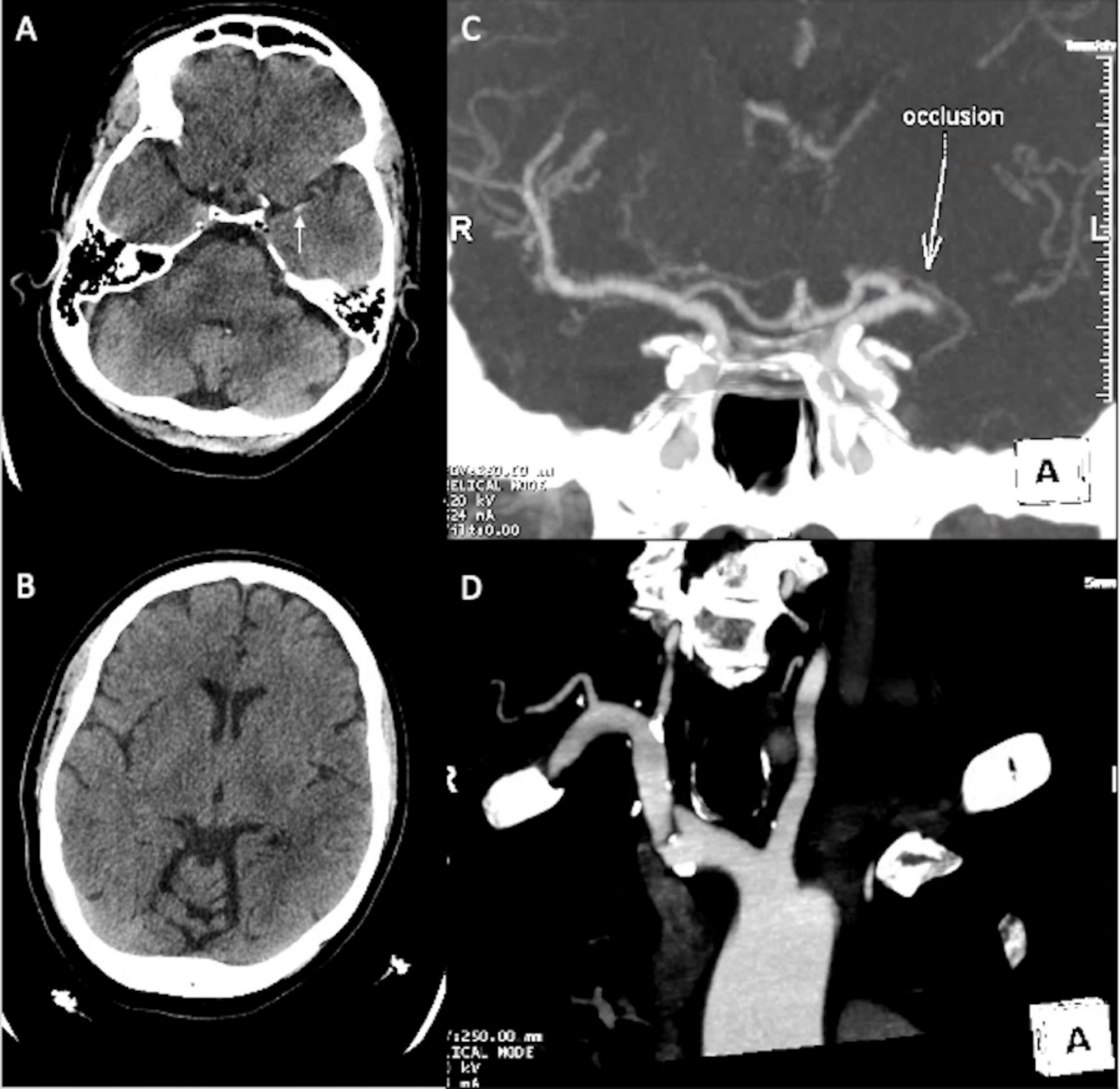
Case 1: neurological imaging. (A) CT of the head shows a left MCA hyperdense sign (white arrow). (B) CT of the head shows no wide extension of left MCA territory infarct. (C) CTA of the head shows the location of MCA occlusion (white arrow). (D) Aortic arch and vessel tortuosity. MCA, middle cerebral artery; CTA, CT angiography

A 74-year-old woman with a medical history of atrial fibrillation on warfarin presented with severe right sided weakness and difficulty speaking. A non-contrast head CT demonstrated a dense left middle cerebral artery (MCA) sign, with CT angiography (CTA) of the head confirming a left M1 occlusion and adequate collaterals (Figure 1). Femoral access was obtained; however, left carotid access was not achieved due to tortuosity of the arch and vessels, leading to femoral access being abandoned. The right radial artery was accessed through a 21-gauge needle allowing placement of a 6-French sheath. Two passes with the Solitaire™ stent-retriever (Medtronic, Minneapolis, MN, USA) achieved TICI 3 revascularization within the left MCA territory. Post-thrombectomy, the patient had hemorrhagic conversion of the infarct with intraparenchymal hemorrhage into the left basal ganglia and extension into the ventricles without hydrocephalus. She was monitored closely but remained stable, and showed improvement of right-sided strength.

Case 2

**Figure 2:**
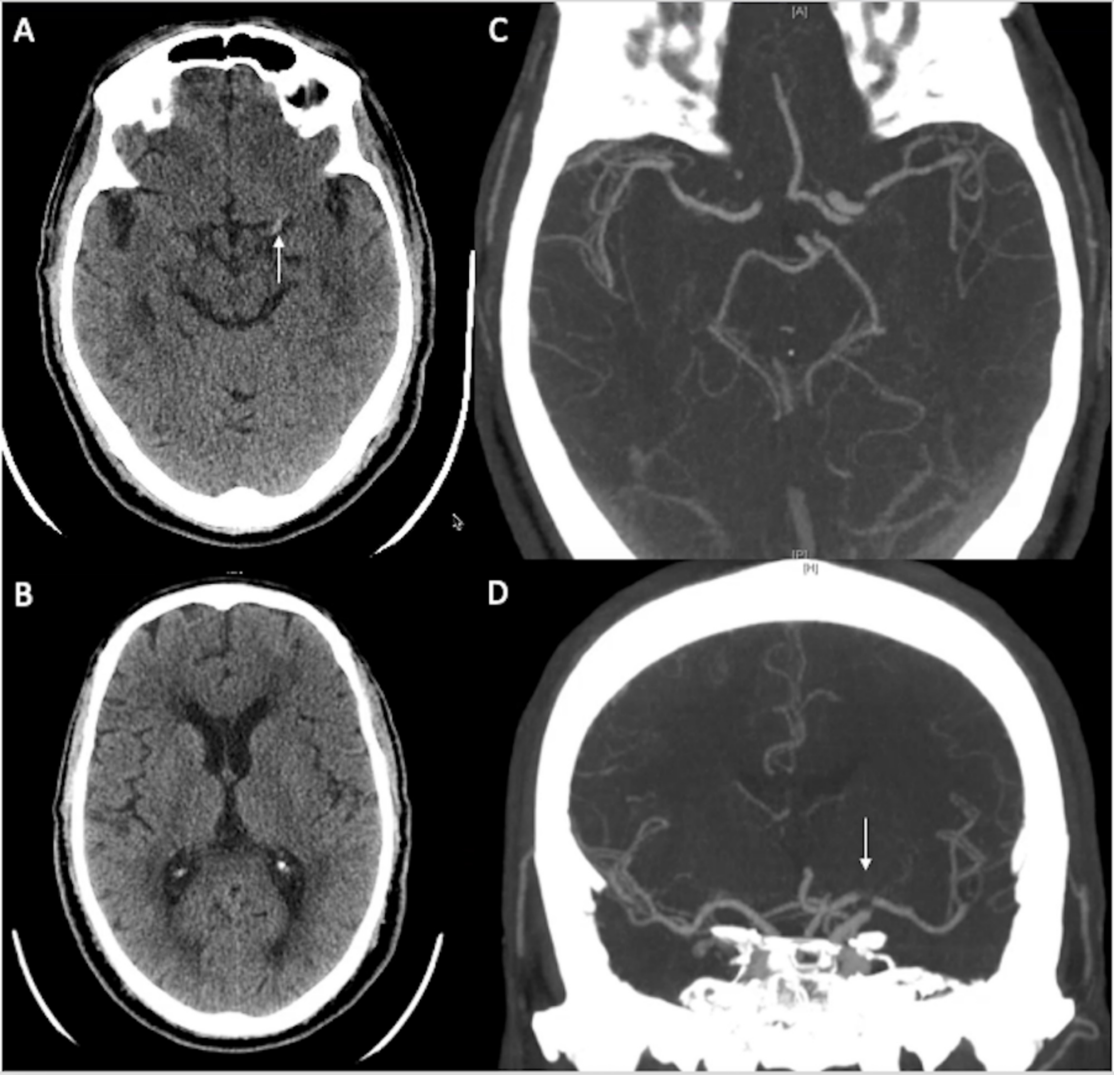
Case 2: neurological imaging. (A) CT of the head shows a left MCA hyperdense sign (white arrow). B) CT of the head shows no wide extension of left MCA territory infarct. (C, D) CTA of the head shows the location of left MCA occlusion. MCA, middle cerebral artery; CTA, CT angiography

A 65-year-old man with history of a type B Aortic dissection that required emergent femoral-femoral bypass with left calf fasciotomy for left leg ischemia presented initially with chest and abdominal pain and was found to have a new type A aortic dissection requiring emergent repair with replacement of ascending aorta and arch. On post-operative day #5, he was found to have new right-sided paralysis and inability to communicate. Initial CT showed a dense left MCA sign. A left ICA partial occlusion was confirmed on CTA and proceeded with thrombectomy (Figure 2). Due to the dissection and femoral popliteal grafts, the common femoral artery could not be used, and thus direct left carotid access was performed under ultrasound. A Solitaire stent-retriever (6x30) was deployed and achieved TICI 2b recanalization with a single pass. Post-thrombectomy, the patient regained some movement on the right with residual aphasia,

## Results

Eleven studies with 51 patients were included in the meta-analysis (all observational cohorts). Patient age ranged from 4th to 10th decade of life (average: 9.3 decade), and 40.1% received IV tissue plasminogen activator (tPA). Initial National Institutes of Health Stroke Scale (NIHSS) score ranged from 1 to 36 (average 17.6). Of the 51 patients, 39 (76%) patients suffered from anterior circulation LVOs versus 12 (24%) from posterior circulation. Site of access included 26 (51%) radial artery punctures, 23 (45%) direct percutaneous cervical carotid punctures, 1 brachial artery puncture, and 1 direct extradural vertebral artery puncture (Tables 1, 2).

**Table 1:** Average of demographics, admission status, procedure-related factors, and imaging and clinical outcomes. *Altschul's cases represent our institutional unpublished data. tPA, tissue plasminogen activator; NIHSS, National Institutes of Health Stroke Scale; TICI, thrombolysis in cerebral infarction; mRS, modified Rankin Scale; M, male; R, right; CCA, common carotid artery; F, female; L, left; ICA, internal carotid artery

Author	Decade of life	Sex	tPA given	Initial NIHSS score	Clot location	Alternative access	TICI score	Access to recanalization (seconds)	mRS at follow-up	Femoral access attempt
Altschul*	7	M	-	23	R M1	R CCA	2b	129	6	+
Altschul	9	M	+	17	R M2	R radial	0		4	-
Altschul	8	F	-	19	L M1	R radial	3	84	4	+
Altschul	7	M	-	21	L ICA	L ICA	2b	31	2	+
Altschul	9	F	+	24	L M2	L CCA	2a	58	6	-
Altschul	7	M	+	25	Basilar	R radial	3	56	1	+
Altschul	4	M	-	25	L ICA	L CCA	2b	49	2	-
Altschul	9	F	+	24	L M1	R radial	3	42	5	+
Altschul	9	F	-	13	L M1	L CCA	2b	27	5	+
Altschul	9	F	+	22	L M1	R radial	3	23	2	-
Altschul	8	F	+	7	Basilar	R radial	2b	43		-
Jadhav et al. [2]	8		-	10	L M1	L CCA	2b	19	4	+
Jadhav et al. [2]	8		-	27	L M1	L CCA	3	7	4	+
Jadhav et al. [2]	6		-	9	L M1	L CCA	2	43	4	+
Jadhav et al. [2]	7		-	22	L ICA	L CCA	2a	30	6	+
Jadhav et al. [2]	7		+	17	L M1	L CCA	2b		0	+
Jadhav et al. [2]	8		-	20	L M1	L CCA	2b	40	2	-
Jadhav et al. [2]	8		-	21	L M1	L CCA	3	8	4	+
Sur et al. [3]	10	F	+	18	L M1	R radial	3	76		+
Sur et al. [3]	9	F	-	8	R M1	R radial	2b	61		+
Sur et al. [3]	9	M	+	18	R M1	R radial	3	29		+
Sur et al. [3]	8	F	+	20	R M1	R radial	3	69		
Sur et al. [3]	10	F	+	12	R ICA	R radial	3	23		
Sur et al. [3]	10	M	+	25	L M1	R radial	3			
Sur et al. [3]	7	M	+	3	R M1	R radial	2b	65		
Sur et al. [3]	10	F	-	20	R M1	R radial	2b	55		
Sur et al. [3]	9	M	+	15	R M2	R radial	3	85		
Sur et al. [3]	10	M	-	1	L M1	R radial	2a			
Sur et al. [3]	9	F	+	24	L M1	R radial	2b	48		
Reznik et al. [4]			-	10	R M1	Radial	3		0	-
Reznik et al. [4]			-	10	R M1	Brachial	2b		1	-
Oselkin et al. [5]	6	F	-	11	Basilar	L CCA	2b	16		-
Oselkin et al. [5]	8	M	+	11	Basilar	Radial	1	53		-
Oselkin et al. [5]	7	M	-	31	Basilar	Radial	3	24		-
Oselkin et al. [5]	7	M	-	5	Basilar	Radial	2b	90		-
Oselkin et al. [5]	7	M	-	30	Basilar	Radial	2b	19		-
Oselkin et al. [5]	9	F	-	8	Basilar	Radial	2b	60		-
Oselkin et al. [5]	9	F	-	22	Basilar	Radial	3	30		-
Oselkin et al. [5]	7	F	-	36	Basilar	Radial	2b	8		-
Oselkin et al. [5]	6	M	-	31	Basilar	Radial	3	22		-
Wiesmann et al. [6]	8		-	23	R ICA	R CCA	3	23	5	+
Wiesmann et al. [6]	4		-	7	R M1	R CCA	3	17	0	-
Wiesmann et al. [6]	8		+	23	R M1	R CCA	2b	19	5	-
Wiesmann et al. [6]	8		-	23	R M1	R CCA	3	21	5	-
Wiesmann et al. [6]	7		+	10	R M1	R CCA	2b	26	1	-
Wiesmann et al. [6]	5		+	10	L M2	L CCA	3	15	1	+
Castaño et al. [7]	9	F	-	11	L M1	L CCA	3		0	+
Mokin et al. [8]				14	R M1	CCA	3	25		-
Mokin et al. [8]				12	R M1	CCA	0			-
Desai et al. [9]			+	25	R vertebral	R vertebral				+
Roche et al. [10]	6	F	-	23	R M1	R CCA	3		0	-

 

**Table 2:** Frequency of cases included in the meta-analysis. NIHSS, National Institutes of Health Stroke Scale; L, left; ICA, internal carotid artery; R, right; CCA, common carotid artery

Clot location	Frequency	Percentage	Alternative access site	Frequency	NIHSS category	Frequency	Percentage
L ICA	3	6%	Brachial	1	0-4	2	4%
R ICA	2	4%	CCA	2			
L M1	15	29%	L CCA	13	16-20	8	16%
L M2	2	4%	L ICA	1			
R M1	15	29%	R CCA	7			
R M2	2	4%	R radial	17	21-42	22	43%
Vertebral	1	2%	R vertebral	1			
Basilar	11	22%	Radial	9			

The average time from symptom onset to first pass/recanalization was 387.5 seconds (n = 33; 95% CI: 289.1-486.0). The average puncture to recanalization time was 40.7 minutes (n = 41; 95% CI: 32.7-49) (Table 3). Technical success defined as a TICI score of 2b or greater was achieved in 43/51 (84%) of patients. For patients with reported outcomes data, average mRS at discharge was 2.93 (n = 26). There were no complications in 25 patients who underwent radial arterial access, and 2/27 cervical access patients developed hematoma, with one managed conservatively and the other surgically.

**Table 3:** Mean time from symptom onset and access to recanalization.

Variable	n	Mean time(seconds)	95% CI
Access to recanalization	41	40.7	32.3- 49.0
Symptom onset to first pass/recanalization	33	387.5	289.1-486.0

## Discussion

TFA for mechanical thrombectomy has become the standard endovascular approach to large vessel AIS, yet often under certain anatomical configurations it can prove difficult and can delay recanalization, which leads to less favorable outcomes [11]. A 2013 study of 130 patients with anterior circulation occlusion showed that 5.1% of patients could not be successfully catheterized using TFA [11]. Patients were grouped into quartiles based on time from groin puncture to target carotid catheterization. Patients in quartile 4 were considered to have difficult carotid access (>30 minutes) and were found to have lower rates of recanalization (TICI ≥ 2a; 60.7% vs. 82.4%; p = 0.02) and less favorable long-term outcome (mRS < 3 at 3 months; 13.6% vs. 41.3%; p = 0.04). It has been well demonstrated that the length of onset to reperfusion time is positively correlated with morbidity and mortality [12-16].

Complex vascular anatomy including aortic arch anatomy, vessel elongation, vessel tortuosity, aneurysmal disease, dissecting disease, peripheral vascular disease, previous vascular surgery and patient height make TFA time consuming and difficult, if not impossible [2-4]. Oftentimes, the delay in gaining access to attempt thrombectomy is due to arch tortuosity [17-19] or conditions that increase the risk of intra-/post-procedural morbidity such as existing aortic dissection [4].

Extra-femoral access (EFA) (i.e., radial, brachial, cervical carotid, or vertebral arteries) provides an alternative method of access for the endovascular treatment of AIS. Although the need for EFA due to TFA failure or difficult vascular anatomy that precludes TFA is relatively uncommon, with figures ranging from 1.5% to 5.1%, EFA can potentially reduce onset-to-reperfusion times once mastered and warrants further investigation. Recent research has focused on the feasibility and efficacy of EFA in AIS in the setting of complex vascular anatomy. However, EFA is usually sought as rescue measures only after TFA fails [2,18], which may cause significant delays in clot removal.

The benefits of EFA (i.e., radial, brachial, cervical carotid, or vertebral arteries) for mechanical thrombectomy have not been well studied, but limited data have supported these approaches since the mid-20th century. Cerebral angiography in the 1950s was originally performed through extra-femoral approaches, specifically direct puncture of the cervical carotid, brachial, and vertebral arteries [19-21]. In the 1960s, trans-femoral catheter placement was introduced and began replacing direct sticks for cerebral angiography [19, 20]. This became popular as it was a one-puncture method that allowed access to all four major cerebral arteries [19,20].

Transradial access

As cerebral angiography has progressed over the past few decades, devices have become more pliable and smaller, and clinicians are no longer restricted to TFA. The trans-radial technique has been well validated in the cardiac literature, with trans-radial access (TRA) for both coronary artery diagnostic and interventional procedures and carotid artery stent procedures as a preferred method or equal alternative [4].

TRA is shown to be successful and efficacious over a range of case series and retrospective analyses [17]. Indeed, meta-analyses examining the reported data for radial access for coronary artery procedures found fewer vascular access complications, lower all-cause mortality, and shorter length of hospital stay compared with traditional TFA [22-27].

In RIVAL (RadIal Vs femorAL Access for Coronary Intervention), a large RCT published in Lancet in 2011, Jolly et al. proposed that there were a lower number of vascular complications in patients who underwent radial access for percutaneous coronary intervention, with 42 post-operative hematomas in 3,507 patients in the radial group compared with 106 in 3,514 patients in the femoral group (hazard ratio: 0.40; 95% CI: 0.28-0.57; p < 0.0001). Their data demonstrate the safety and benefit of this access method [27]. Other added benefits with TRA include the facility of post-procedure hemostasis, shorter patient recovery leading to immediate ambulation and decreased procedure-related costs, increased patient satisfaction, and advantage in obese populations due to larger soft tissue planes for access [5].

Several studies have investigated the claim initially reported that radial access carries an increased risk of embolic events; however, pooled data from >24,000 patients in RCTs and >475,000 patients fail to prove any significant increase in risk ratio (RR) for (any) stroke (RR: 0.87; 95% CI: 0.58-1.29) [28], which is also supported by other reviews [29]. The one significant downside to radial access is the necessity for higher technical skills [22,24]. Another hidden complication includes asymptomatic radial occlusion; however, this is clinically insignificant given the pre-access testing.

Studies included in the meta-analysis

In the field of interventional neurosurgery, extra-femoral approaches have been re-described since the 2000s, specifically using the radial artery [30]. These studies are mainly focused on the treatment of intracranial lesions such as aneurysms and atherosclerotic stenosis. Prior data on EFA have been limited and have mostly been published in the form of retrospective chart reviews, case reports, and case series.

A recent retrospective cohort of 15 (1.5%) out of 1,001 thrombectomy patients were identified as having undergone TRA: 12 of these cases converted mid-procedure to TRA due to TFA failure; in 3, TRA was chosen as a primary strategy [18]. Reperfusion (modified TICI [mTICI] ≥ 2b) was achieved in 9 (60%) of 15 patients [18]. There were no radial puncture site complications noted. At 90 days, two (13%) patients had a good clinical outcome (mRS < 2). Another study examined the technical feasibility of TRA in 11 patients: TRA was chosen as the primary access site for 8 patients and as a secondary measure in 3 patients [2]. Revascularization (mTICI ≥ 2b) was achieved in 10 (91%) of 11 cases, and there were no complications. Certain lesion locations qualify as ideal for TRA, such as posterior circulation thrombosis medial oriented vertebral artery origins. Oselkin et al. contend that initial attempts to access point in such patients should be trans-radial, citing the ease of entry into the vertebral artery on the side of the lesion [5]. They treated nine reported cases of acute basilar artery occlusion (BAO), of which eight achieved recanalization (mTICI ≥ 2b), and there were no complications due to radial artery access. Co-existing conditions such as aortic dissection can also further jeopardize worsening of morbidities and indicate another motive for alternative access. In one study, two such patients were treated with TRA or trans-brachial access and had successful recanalization (mTICI ≥ 2b), clinical improvement, and no procedure-related complications [4].

In our center’s data, TRA was used in six patients (three patients underwent initial attempt at TFA and three underwent access using the primary approach). 5/6 patients achieved successful revascularization (mTICI ≥ 2b). The one patient in whom revascularization was unsuccessful did not represent a procedural access failure, but rather the physical limitations of the catheter system’s length were insufficient to traverse the occlusion. We had no direct post-procedural complications.

There have been other studies using TRA; of note, one large study with 148 angiograms for mechanical thrombectomy attests further to the safety and efficacy of the technique [24].

Trans-cervical access

Examining the trans-cervical carotid access route (TCCA), there are several studies reporting success using this technique in AIS patients with LVO included here. One recent case series identified seven patients in which TCCA was performed [2]. In six of the patients, TFA was attempted as the primary access modality. In one patient, TCCA was initially attempted due to extreme vessel tortuosity visualized on CTA. Six of the seven patients achieved TICI 2b-3 recanalization; there was one patient who developed a post-operative complication (neck hematoma), which led to elective intubation but without the need for surgical removal. In this series, once trans-cervical access was achieved, recanalization was observed at 25 ± 14 minutes. In another retrospective review of a prospectively maintained registry, six patients were identified who underwent acute endovascular thrombectomy through surgical access to the carotid artery, with successful recanalization (TICI ≥ 2b) achieved in all patients (100%) [6]. Recanalization was achieved within 19 ± 5 minutes after establishing carotid access. There was one surgical complication, a small neck hematoma, which was surgically removed without further complication. Additionally, TCCA with TICI 3 reperfusion has been described in AIS patients with difficult access due to tortuosity of vessels and irregular aortic arch in a few case reports, both of which had no reported complications [7,8,18]. There is even a case report of a successful (TICI 3), uncomplicated direct vertebral artery puncture for a BAO performed after both TFA and TRA failed [9].

In our study, TCCA was used in five patients and primary strategy in two patients. TICI 2b revascularization was achieved in two patients, and TICI 2a in the 3rd patient. Four out of five patients achieved successful revascularization (mTICI ≥ 2b), with the exception being a distal occlusion case (left M2) that only achieved TICI 2a recanalization. There were no immediate post-procedural complications.

Significant outcomes and findings

Overall, patients who undergo thrombectomy have guarded prognosis depending on the clot location. Not all studies described reported long-term clinical outcomes; however, the data from our center compares with an average mRS at a follow-up of 3.6 ± 0.9. Two patients died from cardiopulmonary arrest, and one returned to hospital with hemorrhagic conversion.

Our indications prompting EFA were similar to those of the described studies. The most common reason for EFA was a difficult aortic arch followed by vessel tortuosity. There was also one patient with bilateral CFA occlusion and one patient with aortic dissection. A recent retrospective study examining reasons for reperfusion failure in thrombectomy identified that failure to access was the culprit as often as failure to re-establish flow and called for systematic reporting standards for access failure to guide priorities for technical development [6].

This meta-analysis, which combines our center’s case series and the other data identified, demonstrates the safety of EFA including both TRA and trans-cervical access. While the groin is a compressible site with less severe complications compared with carotid or vertebral artery injuries, complications from EFA remain minimal. There were no reported post-procedural complications with cases using TRA, and only 2/27 focal neck hematoma resulted from the TCA cases, one of which resolved with conservative management and the other without compromise of nearby structures through surgical treatment. Both in our center’s data and the overall meta-analysis data show that both procedural success and length of procedures were preserved using EFA techniques, especially as many of these patients may not have been accessible otherwise.

The trans-femoral approach has been associated with increasingly fast rates of groin access to recanalization, particularly with the advent of stent-retrievers. A recent comparison of various endovascular modalities revealed the time from groin puncture to recanalization of 36 ± 18 minutes [16]. Comparable, if not better, rates have been achieved after access, with trans-cervical recanalization of MCA LVO observed at 25 ± 14 minutes [2]. These investigators argued that the proximal access saved time by not only bypassing the tortuous arch but also establishing better support and ease of access to the lesion, and added manual aspiration could also aid in a stronger aspiration effect given the proximal support [2]. This was similarly supported for posterior circulation LVO in this analysis; however, the efficiency data for cervical access are incomplete as many of the reported studies are incomparable due to the mixed use of thrombectomy methods and failure to report exact time marks.

Overall, TRA provides an easy method for bovine arch left ICA LVO or posterior circulation. However, trans-cervical access carries a risk of clinically significant complications if there is post-procedural hemorrhage and there are no ideal closure options [10].

Limitations

The limitation of this study and previous studies is that the majority of cases in which EFA was used, it was attempted after TFA failed initially. Therefore. time to recanalization will be high, and outcomes will likely be poor in this cohort. There may be a subset of patients that could benefit from an initial extra-femoral approach in order to reduce time to recanalization. Further research is warranted to further delineate who these patients are. Likely, patients who could benefit would include those with type 2 or 3 aortic arches with proximal tortuosity, or patients with bovine-type arches for left-sided lesions.

## Conclusions

We summarize the safety and effectiveness of alternative access in interventional AIS treatment. Further standardization of these techniques, guidelines to prospectively identify individual patient need for EFA, and development of devices tailored for trans-radial and trans-cervical carotid approaches can become useful adjuncts to neurointerventionalists, which can further advance the field of interventional stroke treatment. Development of protocols for early triage and identification of patients who would benefit from EFA as the primary strategy would likely lead to better clinical outcomes.

## References

[1] Powers WJ, Rabinstein AA, Ackerson T (2018). 2018 guidelines for the early management of patients with acute ischemic stroke: a guideline for healthcare professionals from the American Heart Association/American Stroke Association. Stroke.

[2] Jadhav AP, Ribo M, Grandhi R, Linares G, Aghaebrahim A, Jovin TG, Jankowitz BT (2014). Transcervical access in acute ischemic stroke. J Neurointerv Surg.

[3] Sur S, Snelling B, Khandelwal P, Caplan JM, Peterson EC, Starke RM, Yavagal DR (2017). Transradial approach for mechanical thrombectomy in anterior circulation large-vessel occlusion. Neurosurg Focus.

[4] Reznik ME, Espinosa-Morales AD, Jumaa MA, Zaidi S, Ducruet AF, Jadhav AP (2017). Endovascular thrombectomy in the setting of aortic dissection. J Neurointerv Surg.

[5] Oselkin M, Satti SR, Sundararajan SH, Kung D, Hurst RW, Pukenas BA (2018). Endovascular treatment for acute basilar thrombosis via a transradial approach: Initial experience and future considerations. Interv Neuroradiol.

[6] Wiesmann M, Kalder J, Reich A, Brockmann M-A, Othman A, Greiner A, Nikoubashman O (2016). Feasibility of combined surgical and endovascular carotid access for interventional treatment of ischemic stroke. J Neurointerv Surg.

[7] Castaño C, Remollo S, García MR, Hidalgo C, Hernández Hernández, Perez M, Ciorba M (2015). Mechanical thrombectomy with “ADAPT” technique by transcervical access in acute ischemic stroke. Neuroradiology.

[8] Mokin M, Snyder KV, Levy EI, Hopkins LN, Siddiqui AH (2015). Direct carotid artery puncture access for endovascular treatment of acute ischemic stroke: technical aspects, advantages, and limitations. J Neurointerv Surg.

[9] Desai JA, Almekhlafi MA, Hill MD, Goyal M, Eesa M (2014). Ultrasound guided V3 segment vertebral artery direct percutaneous puncture for basilar artery mechanical thrombectomy in acute stroke: a technical report. Case Reports.

[10] Roche AD, Murphy B, Adams N, Sheahan R, Brennan P, Looby S (2017). Direct common carotid artery puncture for endovascular treatment of acute large vessel ischemic stroke in a patient with aortic coarctation. J Stroke Cerebrovasc Dis.

[11] Ribo M, Flores A, Rubiera M (2013). Difficult catheter access to the occluded vessel during endovascular treatment of acute ischemic stroke is associated with worse clinical outcome. J Neurointerv Surg.

[12] Hassan AE, Chaudhry SA, Miley JT, Khatri R, Hassan SA, Suri MFK, Qureshi AI (2013). Microcatheter to recanalization (procedure time) predicts outcomes in endovascular treatment in patients with acute ischemic stroke: when do we stop?. Am J Neuroradiol.

[13] Khatri P, Abruzzo T, Yeatts SD, Nichols C, Broderick JP, Tomsick T (2009). Good clinical outcome after ischemic stroke with successful revascularization is time-dependent. Neurology.

[14] Mazighi M, Chaudhry SA, Ribo M (2013). Impact of onset-to-reperfusion time on stroke mortality: A collaborative pooled analysis. Circulation.

[15] Spiotta AM, Vargas J, Turner R, Chaudry MI, Battenhouse H, Turk AS (2014). The golden hour of stroke intervention: effect of thrombectomy procedural time in acute ischemic stroke on outcome. J Neurointerv Surg.

[16] Ribo M, Molina CA, Jankowitz B (2014). Stentrievers versus other endovascular treatment methods for acute stroke: comparison of procedural results and their relationship to outcomes. J Neurointerv Surg.

[17] Kaymaz Z, Nikoubashman O, Brockmann M, Wiesmann M, Brockmann C (2017). Influence of carotid tortuosity on internal carotid artery access time in the treatment of acute ischemic stroke. Interv Neuroradiol.

[18] Haussen DC, Nogueira RG, DeSousa KG (2016). Transradial access in acute ischemic stroke intervention. J Neurointerv Surg.

[19] Gould PL, Peyton WT, French LA (1955). Vertebral angiography by retrograde injection of the brachial artery. J Neurosurg.

[20] Kuhn RA (1959). Brachial cerebral angiography. J Neurosurg.

[21] Leeds NE, Kieffer SA (2000). Evolution of diagnostic neuroradiology from 1904 to 1999. Radiology.

[22] Agostoni P, Biondi-Zoccai GGL, De Benedictis ML (2004). Radial versus femoral approach for percutaneous coronary diagnostic and interventional procedures: Systematic overview and meta-analysis of randomized trials. J Am Coll Cardiol.

[23] Campeau L (1989). Percutaneous radial artery approach for coronary angiography. Cathet Cardiovasc Diagn.

[24] Snelling BM, Sur S, Shah SS (2018). Transradial cerebral angiography: techniques and outcomes. J Neurointerv Surg.

[25] Eichhöfer J, Horlick E, Ivanov J (2008). Decreased complication rates using the transradial compared to the transfemoral approach in percutaneous coronary intervention in the era of routine stenting and glycoprotein platelet IIb/IIIa inhibitor use: a large single-center experience. Am Heart J.

[26] Ferrante G, Rao S V, Jüni P (2016). Radial versus femoral access for coronary interventions across the entire spectrum of patients with coronary artery disease: a meta-analysis of randomized trials. JACC Cardiovasc Interv.

[27] Jolly SS, Yusuf S, Cairns J (2011). Radial versus femoral access for coronary angiography and intervention in patients with acute coronary syndromes (RIVAL): a randomised, parallel group, multicentre trial. Lancet.

[28] Sirker A, Kwok CS, Kotronias R (2016). Influence of access site choice for cardiac catheterization on risk of adverse neurological events: a systematic review and meta-analysis. Am Heart J.

[29] Ratib K, Mamas MA, Routledge HC, Ludman PF, Fraser D, Nolan J (2013). Influence of access site choice on incidence of neurologic complications after percutaneous coronary intervention. Am Heart J.

[30] Bendok BR, Przybylo JH, Parkinson R, Hu Y, Awad IA, Batjer HH (2005). Neuroendovascular interventions for intracranial posterior circulation disease via the transradial approach: technical case report. Neurosurgery.

